# Probing the Mode of Antibacterial Action of Silver Nanoparticles Synthesized by Laser Ablation in Water: What Fluorescence and AFM Data Tell Us

**DOI:** 10.3390/nano10061040

**Published:** 2020-05-29

**Authors:** Lucija Krce, Matilda Šprung, Tomislav Rončević, Ana Maravić, Vedrana Čikeš Čulić, Damjan Blažeka, Nikša Krstulović, Ivica Aviani

**Affiliations:** 1 Department of Physics, Faculty of Science, University of Split, Ruđera Boškovića 33, 21000 Split, Croatia; iaviani@pmfst.hr; 2 Department of Chemistry, Faculty of Science, University of Split, Ruđera Boškovića 33, 21000 Split, Croatia; msprung@pmfst.hr; 3 Department of Biology, Faculty of Science, University of Split, Ruđera Boškovića 33, 21000 Split, Croatia; troncevic@pmfst.hr (T.R.); amaravic@pmfst.hr (A.M.); 4 Department of Medical Chemistry and Biochemistry, School of Medicine, University of Split, Šoltanska ulica 2, 21000 Split, Croatia; vedrana.cikes.culic@mefst.hr; 5 Institute of Physics, Bijenička cesta 46, 10000 Zagreb, Croatia; dblazeka@ifs.hr (D.B.); niksak@ifs.hr (N.K.)

**Keywords:** laser-synthesized nanoparticles, silver nanoparticles, mode of antibacterial action, nano-bio interactions, reactive oxygen species, atomic force microscopy, Young modulus

## Abstract

We aim to elucidate the mode of antibacterial action of the laser-synthesized silver colloid against *Escherichia coli*. Membrane integrity was studied by flow cytometry, while the strain viability of the treated culture was determined by plating. The spectrofluorometry was used to obtain the time development of the reactive oxygen species (ROS) inside the nanoparticle-treated bacterial cells. An integrated atomic force and bright-field/fluorescence microscopy system enabled the study of the cell morphology, Young modulus, viability, and integrity before and during the treatment. Upon lethal treatment, not all bacterial cells were shown to be permeabilized and have mostly kept their morphology with an indication of cell lysis. Young modulus of untreated cells was shown to be distinctly bimodal, with randomly distributed softer parts, while treated cells exhibited exponential softening of the stiffer parts in time. Silver nanoparticles and bacteria have shown a masking effect on the raw fluorescence signal through absorbance and scattering. The contribution of cellular ROS in the total fluorescence signal was resolved and it was proven that the ROS level inside the lethally treated cells is not significant. It was found that the laser-synthesized silver nanoparticles mode of antibacterial action includes reduction of the cell’s Young modulus in time and subsequently the cell leakage.

## 1. Introduction

Silver nanoparticles (AgNPs) have retained the interest of the scientific community, mostly due to their outstanding antibacterial property. They have been proven to have a synergistic effect on the antibacterial potential of antibiotics [1,2] and are increasingly being used for antimicrobial coating [3]. These promising results emphasize the need for thorough characterization of the used AgNPs and understanding of their antibacterial mode of action.

As suggested in the recent review paper [4], there are two main hypotheses for their antibacterial mode of action. The first hypothesis relies on the nanoparticle’s electrostatic interaction with the bacterium membrane and/or with membrane proteins which leads to membrane’s partial dissolution. The second hypothesis presumes penetration of the AgNPs inside the cell/membrane and subsequent release of silver ions, followed by the increase of the reactive oxygen species (ROS) and cell oxidative stress. The two scenarios do not need to be mutually exclusive [4]. Ivask et al. [5] emphasize the role of the dissolved silver ions in the toxicity mechanism, and also the importance of AgNPs’ physicochemical surface properties which govern the interaction with the bacterial cells. For coated silver nanoparticles, antimicrobial effects were found to be the interplay of nanoparticle (NP) size, solubility, and surface coating [6]. The activity of transition metals, such as silver, should also be considered to be the possible reason for potent antibacterial activity. Transition metals have an affinity to associate with R-SH groups, which can disrupt the function of specific enzymes or disrupt S–S bridges necessary to maintain the integrity of folded proteins, causing detrimental effects to the metabolism and the physiology of the cell [7]. Particle-specific antibacterial potential, which is manifested through increased intracellular concentration of Ag ions, has been previously reported as the AgNPs’ main mechanism to battle bacteria [8]. Xiu et al. [9] report that the antimicrobial activity of AgNPs is solely due to the release of Ag ions. Notably, Durán et al. [10] state that the AgNPs exhibit their antibacterial activity through the cell’s membrane disruption and DNA transformation via ROS but that there is a lack of data regarding the temporal resolution of the ROS production or possible membrane alterations induced by the AgNPs. Taken together, it is reasonable to say that the AgNPs adhesion to and/or penetration inside the cells, ROS generation and modulation of microbial signal transduction pathways are recognized as the most notable modes of antimicrobial action [11].

In comparison, AuNPs [12] and AuPtNPs [13] do not induce the generation of ROS. On the other hand, the antibacterial mechanism of copper-bearing titanium alloys includes an increased amount of ROS, among other cells’ alterations [14]. The toxicity of graphene oxide towards bacteria may be attributed to both membrane and oxidative stress [15], while ZnO NPs induce ROS production and TiO_2_ NPs promote changes in the outer membrane proteins [16]. 

Most of the reports concerning the antibacterial effect of the AgNPs refer to colloids produced by the reduction of metal salts with a chemical agent which results in colloids containing unwanted chemical byproducts. There are eco-friendly alternatives such as biosynthesis with bacteria, fungi, or plant-related parts [17]. However, one of the most convenient techniques is the physical production of colloids by using laser ablation in liquids (LAL) [18]. This method enables the production of pure colloids without byproducts and with unique surface properties [19] which is extremely important when one is trying to elucidate their mode of antibacterial action.

Microscopy techniques are rather useful since they can reveal membrane alteration caused by the AgNPs treatment. Previously, electron-microscopy data have revealed gaps in the cells’ membrane and disorganization of entire cells [20], nanoparticles accumulated in the membrane, penetration into the cells and leaking of intracellular substances [21], nanoparticles found on cells’ surface and attached to the substance released by the cells [22]. Transmission electron-microscopy images of *Escherichia coli* (*E. coli*) treated with LAL-produced AgNPs revealed that the nanoparticles have penetrated the cells’ membranes and entered the cells while the cells had irregular appearance [23]. However, this technique does not enable the inspection of cells in their native environment.

Atomic force microscopy (AFM), enables the study of morphology and nanomechanical properties of live bacteria in different media. The loss of cellular morphology of AgNP-treated *Staphylococcus aureus* [24] and alterations in the bacterial cell surface [25] is reported for AFM performed on dried out bacterial cells. A rare report on AFM performed on hydrated AgNP-treated *E. coli* cells reveals membrane rupture, cell stiffness reduction, and appearance of cellular debris around the cells [26]. Although being a very convenient technique, AFM is still under-used in the study of AgNP-treated bacteria and according to our knowledge, there is no time-resolved analysis of Young modulus (YM) of the AgNP-treated bacterial cells.

AgNPs and Ag ions might act as catalysts and increase the generation of ROS which can lead to oxidative stress [19]. ROS detection is mostly performed using the 2′,7′-dichlorodihydrofluorescein (DCFH_2_) which oxidizes inside the cells to yield the highly fluorescent 2′,7′-dichlorofluorescein (DCF) [27]. The intensity of the fluorescence signal is proportional to the amount of ROS [28]. The research data obtained from previous reports suggest that: treatment with AgNPs alone did not induce significant ROS formation [29]; a significant increase in the DCF fluorescence intensity was observed for AgNP-treated bacterial cells [30]; colloidal silver significantly increased the production of ROS when compared with untreated cells [31]; ROS played a very important role in the antibacterial mechanism of AgNPs [32]. Interestingly, ROS detection data in AgNP-treated *E. coli* [33] showed that the fluorescence signal of AgNP-treated cells is lower than for the non-treated cells, for most treatment concentrations. ROS increase in bacterial cells, induced by biosynthesized AgNPs, was confirmed by DCFH_2_ and antioxidant scavenging [34]. Treatment with the LAL-produced AgNPs resulted in the particle-size dependent ROS generation, highest for particles with an average size of 19 nm [35]. It is also worth noting that no significant difference in silver ion toxicity towards bacteria has been observed between anaerobic and aerobic conditions, which rules out oxidative stress by ROS as an important antibacterial mechanism for silver ions [36].

The AgNPs have been used in industry for food packaging, medical device coating, and environmental sensing [37]. LAL-synthesized AgNPs have already been used to fabricate AgNP-impregnated paper fines sheets with antimicrobial activity [38]. This type of AgNPs might also be used as drug carriers by applying different functionalization strategies [39]. Another application might include AgNP-impregnated polymeric nanofibers [40] for wound healing or the incorporation of AgNPs into a device for delivering bioactive agents, such as protein scaffolds [41]. These possible applications require careful consideration of AgNPs’ biological activity and potential toxicity as well as careful quality, efficacy, and safety evaluation of final AgNP-enabled materials complying with the Safe-by-Design approach [3]. It must be noted that there is a justified global concern regarding the toxicity of AgNPs [42] and engineered nanomaterial in general [43,44].

Altogether, despite the numerous studies, there are still contrary reports on the mode of antibacterial action of colloidal silver. This could be partially related to the difference in the AgNPs colloid properties which depend on the synthesis method. Recently, based on modeling of the growth of AgNP-treated bacteria, we have shown that the antibacterial action of LAL-synthesized AgNPs is closely related to their penetration into the cell [45] which should be supported by other complementary experimental methods found in the literature. All the above encouraged us to extend our research of bactericidal effects induced by LAL-synthesized AgNPs, produced in our lab.

Most of our findings are done by studying the time development of the DCF fluorescence signal, which is proportional to the ROS level, and time-dependent YM spectroscopy of bacterial cells. This paper is aimed to contribute to the understanding of the mode of antibacterial action of AgNPs against *E. coli* as a model organism and to discuss some of the findings given in the literature in the context of our results.

## 2. Materials and Methods

The laser synthesis of AgNPs in water and thorough characterization of the produced colloid is described in [45]. The nanoparticles were determined to be spherical-like with the mean diameter of 13.1 nm (obtained from the transmission electron microscopy (TEM) measurements). The produced colloid was stable with the zeta potential of ξ=–(53.1±1.1) mV and the obtained UV-Vis maximum at 404 nm. The mass concentration of AgNPs in the produced colloid was calculated to be of 220 ± 32 µg/mL. The MIC = MBC value is reported as a volume share *v* of the colloid in the batch culture and was obtained to be v=(30±4)%. The colloid volume share *v* correlates to the mass concentration of AgNPs in the batch when it is multiplied by the mass concentration of the produced colloid. Therefore, in further text the colloid volume share *v* will be referred to as the AgNPs’ concentration.

The *E. coli* DH5α cells, used in this study for all experiments, were grown in the nutritionally impoverished Luria Bertani (LB) medium (5.0 g of tryptone, 2.5 g of yeast extract and 5.0 g of NaCl per 1 L of deionized sterile water). Overnight bacterial culture, grown at 37 °C and 220 rpm, was diluted for at least 15 times in the fresh medium. Cells were further grown for 1.5 h in the same conditions. The minimal inhibitory concentration (MIC) and the minimal bactericidal concentration (MBC) of the produced colloid were determined as previously reported [45]. These experiments were repeated for each colloid production and are reported in terms of volume shares *v* of the colloid in the batch culture.

### 2.1. Time-Killing Assay

The assay was performed as explained in Blažević et al. [46]. Exponentially grown bacterial cells were adjusted spectrophotometrically to a density of 10^6^ CFU/mL and exposed to ½MIC, MIC, and 2MIC values of the produced colloid. The strain viability was determined by plating the serial dilutions after the incubation at 37 °C for 0, 15, 30, 60, 90, 240, 360, and 600 min. Colonies were grown on Mueller Hinton agar (MHA) plates and were counted after 24 h. All measurements were done in duplicates and the reported values are their average.

### 2.2. Membrane Integrity Assay

The effect of the AgNPs on the bacterial inner membrane integrity was studied by measuring the percentage of propidium iodide (PI) positive cells after exposure to nanoparticles, using an Accuri C6 flow cytometer (BD Biosciences, San Jose, CA, USA). Cells penetrated by this stain are often referred to as the PI-positive cells (PI+). Cell staining was done as described previously in Rončević et al. [47]. The cells were treated by AgNPs in concentrations corresponding to ½MIC, MIC, and 2MIC, and the measurements were obtained after 0, 15, 30, 60, and 90 min of the treatment. Melittin, a strong membranolytic peptide [48], was used as a positive control, while stained untreated cells were used as the negative control. Non-stained cells and single stained samples were used to compensate fluorescence channels on the cytometer and to adjust appropriate gates on dot-plots. All measurements were done in triplicates, and for each incubation time at least 10,000 cells were collected. Data analysis was carried out with FlowLogic 6.0 software.

### 2.3. Reactive Oxygen Species (ROS) Detection

The obtained bacterial culture was centrifuged at 4500× *g* for 5 min at room temperature. Pelleted cells were then resuspended (1:10) in the 1x phosphate-buffered saline (PBS) and the colloid AgNPs were added to the culture in several volume shares *v*: 0.01, 0.05, 0.10, 0.20, 0.25 and 0.30. The fluorimetric probe 2′,7′-dichlorodihydrofluorescein diacetate (DCFH_2_-DA) (Sigma-Aldrich, St. Louis, MO, USA) was diluted in dimethyl sulfoxide (DMSO) to the final concentration of 1 mg/mL and 2 μL of diluted dye was added to the vials while the final volume of the reaction mix was 1 mL. After adding the AgNPs, samples were incubated for 30 min with shaking at 37 °C in the dark. The 0.33 mM H*_2_*O*_2_* bacterial treatment was used as a positive control. Finally, 200 μL of the reaction mix was transferred to the microtiter plate (Porvair Science, Wrexham, UK) and the fluorescence signal was obtained every 3 min using the spectrofluorometer (LS 55, Perkin Elmer, Waltham, MA, USA) at the 492 nm excitation and 523 nm emission wavelengths.

To measure the time dependence of the fluorescence signal without the cells, another set of samples was prepared in the same way while cell suspension was replaced by PBS. Also, two control data sets were obtained for the stained non-treated culture suspension and the stained PBS samples. All experiments were done in quadruplicates and the reported values are their averages.

### 2.4. AFM Measurements

A 50 µL aliquot of the prepared culture was applied to the sterile Petri dishes (WPI, Sarasota, FL, USA) coated with the Cell-Tak (Corning, New York, NY, USA) solution prepared as we have previously reported [49]. To eliminate non-attached and loosely attached bacterial cells, the culture was thoroughly rinsed with the sterile medium 10 min after the application. This was done taking care not to dry out the sample during rising.

AFM measurements were carried out using a Nanowizard IV system (JPK/Bruker, Berlin, Germany) operating in the quantitative imaging (QI) mode (force-distance curve-based mode) using the pre-calibrated PFQNM-LC-A-CAL probes (Bruker, Billerica, MA, USA). The setpoint was kept at 1.2 nN while the extend/retract speed was up to 150 µm/s. Each measurement was done with a resolution of 128 × 128 pixels and with 10 μm scan sizes. Several AFM measurements were obtained before the treatment to make sure that the AFM probing does not influence the bacterial viability. The rinsing of the sample with the fresh medium followed this step. AFM probing was then done for cells treated with AgNPs at the concentration corresponding to 2MIC at several time points. All AFM measurements were done in the growth medium at 37 °C. The AFM data processing was carried out by the JPK data processing software. This software allows batch processing of the obtained force-distance curves. The processing was done to obtain the YM of the treated and non-treated bacterial cells for several time points. The YM was calculated by applying the Hertz model to the first 50 nm of the indentation made by the spherical indenter that has an end radius of 65 nm. The distribution of the bacterial YM was obtained from modulus data points whose corresponding height data were in the 5% of the highest points for each scan line. The baseline was taken to be the highest point belonging to the substrate.

### 2.5. Fluorescence and Bright-Field Microscopy

The used AFM system is integrated with the inverted optical fluorescence microscope IX73 (Olympus, Tokyo, Japan). The integration allows the overlay of the bright-field and the AFM image. Bright-field images were taken during the pre-treatment and treatment periods. Fluorescence images were obtained immediately after the final AFM measurements. The growth medium and the colloid mixture were replaced by the physiological saline solution and the cells were stained by the green-fluorescent nucleic stain SYTO 9 and the red-fluorescent nucleic PI (LIVE/DEAD BacLight Bacterial Viability Kit L7012; Invitrogen, Carlsbad, CA, USA). The staining was done by adding 1.5 µL of each dye per mL of the bacterial culture.

## 3. Results

**Figure 1:**
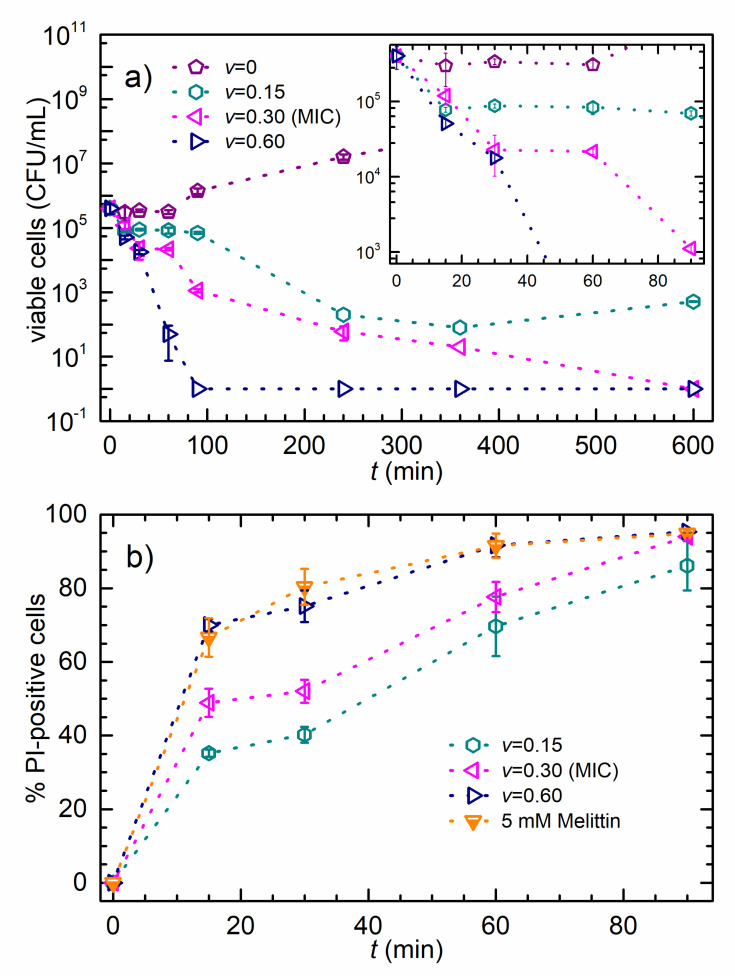
**The time-killing and membrane integrity assays**. (**a**) Viable-cell concentration versus time. The mean values of viable-cell concentration ± SD for *E. coli* incubated with different AgNPs concentrations, as given in the legend. The inset is the first 90 min of the experiment. (**b**) Percentage of PI-positive cells versus time. The data represent the percentage of PI-positive cells ± SD for *E. coli* incubated with different AgNPs concentrations, or with Melittin, as given in the legend.

Results of the time-killing assay are given in Figure 1a where cell count for bacteria treated with different concentrations of AgNPs and for non-treated bacteria are plotted in a logarithmic scale as a function of time. The inset shows the first 90 min of the experiment. The non-treated bacteria exhibit exponential growth, while the viable cells’ data obtained for v=0.15 treatment develop a broad minimum before the growth prevails. The viable cells data obtained for the MIC treatment show a continuous decrease until the complete population inactivation after 600 min of treatment. As expected, the 2MIC (v=0.60) treatment caused much faster viable-cell decrease, and the complete population inactivation occurs after about 100 min of treatment.

The effect of AgNPs on the *E. coli* membrane integrity is shown in Figure 1b. The symbols are the percentages of the PI-positive cells as a function of time upon the produced colloid treatment. The cells were incubated with the AgNPs for 90 min at concentrations of ½MIC (v=0.15), MIC (v=0.30), and 2MIC (v=0.60). Melittin was used as a positive control. The data are expressed as the mean values of percentages of the PI-positive cells ± SD. PI, commonly used fluorescent stain for identifying permeabilized cells in a population, penetrates only cells with disrupted membranes and is generally excluded from viable cells [50]. The increase of the PI signal shows that the treatment of *E. coli* cells with the AgNPs caused significant inner membrane permeability at all tested concentrations (Figure 1b). This effect was dose and time-dependent with ~40% PI-positive cells after 15 min of treatment with AgNPs at ½MIC concentration, increasing to almost 90% after 90 min of treatment. Treatment with the AgNPs at the MIC concentration caused somewhat more damage with 94% PI-positive cells after 90 min of incubation. Interestingly, the treatment with AgNPs at the 2 MIC concentration resulted in a more rapid response, i.e., over 90% of all cells were PI-positive after 60 min of incubation. However, after 90 min of treatment at the 2MIC concentration, the percentage of PI-positive cells did not change compared to treatment with AgNPs at MIC concentration.

Melittin was used as a positive control since it has a strong membranolytic effect [48]. Figure 1b shows that the 2MIC AgNPs and the 5 mM Melittin treatment have almost the same effect on bacterial membrane integrity. After 90 min of treatment with Melittin and AgNPs, most of the cells endured damage and the percentage of the PI+ cells is almost the same, for both MIC and 2MIC treatments.

**Figure 2:**
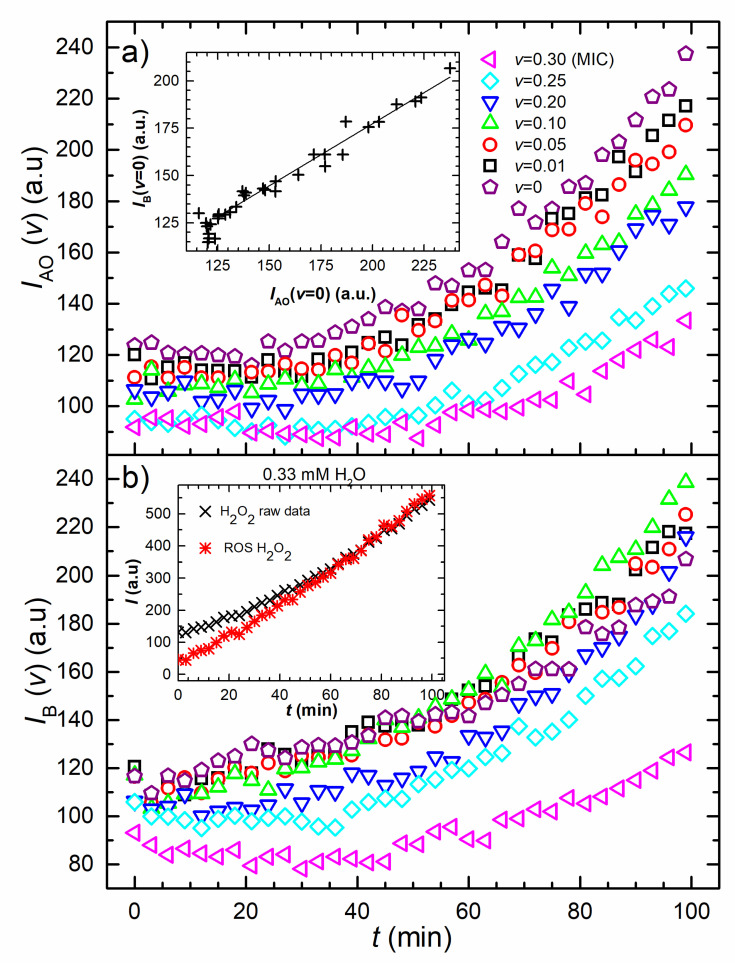
**Evolution of the raw fluorescence data**. (**a**) Raw fluorescence intensity versus time. Time dependence of the fluorescence signal developed when the DCFH_2_-DA dye is diluted in non-inoculated PBS and mixed with the produced colloid. The legend reveals AgNPs concentrations in the mixture. The inset shows the correlation between the non-inoculated and inoculated samples raw fluorescence data that are AgNP-free (*v* = 0) and its linear fit. (**b**) Raw fluorescence intensity versus time. Time development of the fluorescence signal of the same mixture given in (**a**) when inoculated with *E. coli* cells. The cross-shaped symbols in the inset are the raw fluorescence signal of H_2_O_2_ treated *E. coli* cells and the double cross-shaped symbols are the cellular ROS data.

Figure 2 shows the time dependence of the fluorescence signal developed when the DCFH_2_-DA dye is added to samples containing different concentrations of AgNPs and H_2_O_2_. Figure 2a reveals the fluorescence signal obtained for non-inoculated dyed PBS and different AgNPs concentrations mixtures. All curves exhibit an increase in the fluorescence signal. The sample without nanoparticles exhibits the highest signal at all-time points. The signal of each curve reduces as the concentration of the AgNPs increases. Figure 2a shows the mean values of the obtained quadruplet measurements for which the standard deviations were expressed as a percentage of the mean value. The mean values of standard deviations for each data set are: 6.9% (*v* = 0), 5.8% (*v* = 0.01), 7.0% (*v* = 0.05), 6.4% (*v* = 0.10), 7.1% (*v* = 0.20), 6.7% (*v* = 0.25) and 5.0% (*v* = 0.30). The inset shows the correlation between the non-inoculated and inoculated sample without AgNPs. Best linear fit for this correlation gives y=(0.66x+46) a.u., with *R*^2^ = 0.97.

Figure 2b depicts the fluorescence data of the inoculated samples for the same AgNPs concentrations. The highest signal is obtained for v=0.15 while the signals for v=0.05 and v=0.01 treatments are slightly smaller. The fluorescence signal initially decreases for v=0.25 and *v* = (MIC) samples, before the increase prevails. When compared with the fluorescence data given in Figure 2a, the change in the total signal is expected to occur due to the intracellular ROS production, induced by the presence of AgNPs. Interestingly, the final fluorescence signal of inoculated samples is higher than the non-inoculated ones for all samples except two; the MIC-treated and the non-treated sample signal. The mean values of standard deviations for each data set are: 4.6% (*v* = 0), 6.7% (*v* = 0.01), 4.3% (*v* = 0.05), 4.0% (*v* = 0.10), 5.2% (*v* = 0.20), 4.5% (*v* = 0.25) and 5.9% (*v* = 0.30).

H_2_O_2_ is a known oxidizing and antibacterial agent to which the membranes are semi-permeable [51]. The fluorescence results obtained for the bacterial H_2_O_2_ treatment are shown in the inset of Figure 2b. The cross-shaped symbols are the raw fluorescence signal of H_2_O_2_ treated *E. coli* cells and the double cross-shaped symbols are the bacterial ROS fluorescence data, obtained as explained in the Discussion section. The mean value of standard deviations for the H_2_O_2_ raw data was obtained to be 4.2%.

**Figure 3:**
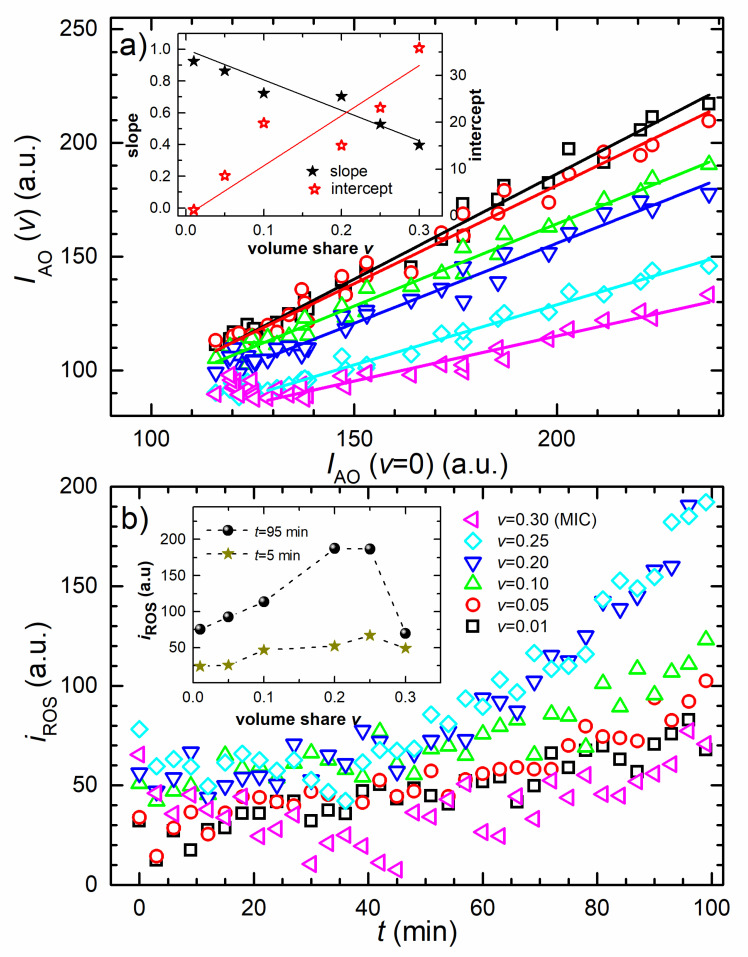
**Fluorescence data analysis**. (**a**) The correlation between *v* = 0 and *v* ≠ 0 fluorescence intensity data from Figure 2a. The symbols are the experimental data and the lines are the best linear fits where the lines’ length denotes the data range selected for each fit. The inset gives the slope (full stars) and the intercept (open stars) of the fits as a function of AgNPs concentration. Each data set is fitted to a line. (**b**) ROS fluorescence intensity versus time. Time evolution of the AgNP-induced bacterial ROS fluorescence (*i*_ROS_) signal extracted from the raw data, as described in the Discussion section. The inset shows the concentration dependence of the *i*_ROS_ average for the first and last three time points. The AgNPs concentration is given in the legend.

The correlations between v=0 and v≠0 fluorescence data of non-inoculated samples are given in Figure 3a. For all concentrations, the linear correlation is found with *R*^2^ ≥ 0.98. Please note that the *v* = MIC, v=0.25, and v=0.20 sample data deviate from linearity for *t* < 36 min. For these concentrations, the linear fit is applied for the *t* ≥ 36 min. The concentration dependencies of the correlations’ coefficient (slope) and the intercept are given in the inset of Figure 3a. The symbols are the data and the full lines are the one-parameter linear fits. The correlations’ slope decreases with the concentration and the best linear fit, with intercept fixed to 1, is found to be y=1−1.92x, with *R*^2^ = 0.99.

The correlations’ intercept increases proportionally with the concentration, and the best proportional fit is found to be y=107x, with *R*^2^ = 0.92.

Figure 3b shows the time evolution of the bacterial ROS signal extracted from the raw data, as explained in the Discussion section. Initially, the signal is higher for higher AgNPs concentrations. It increases in time with the concentration-dependent rate, except for the MIC sample, which initially decreases, and then increases, exhibiting a broad minimum at about 50 min. For the smallest tested AgNPs concentrations (v=0.01, v=0.05), the increase is low but for the higher concentrations (v=0.10, v=0.20, v=0.25) it is rather significant. The highest final ROS signals were obtained for v=0.20 and v=0.25 samples. The inset shows concentration dependency of the ROS signal obtained at the beginning (full stars) and the end (full circles) of the treatment. The values are the signal averages of the three consecutive points. The ROS signal increases proportionally with concentration *v*, for 0.20<v<0.25 assumes the maximal value of about 200 a.u. and then for *v* = 0.30 suddenly drops to about 50 a.u. Notably, the MIC treatment induces a significantly lower ROS level.

**Figure 4:**
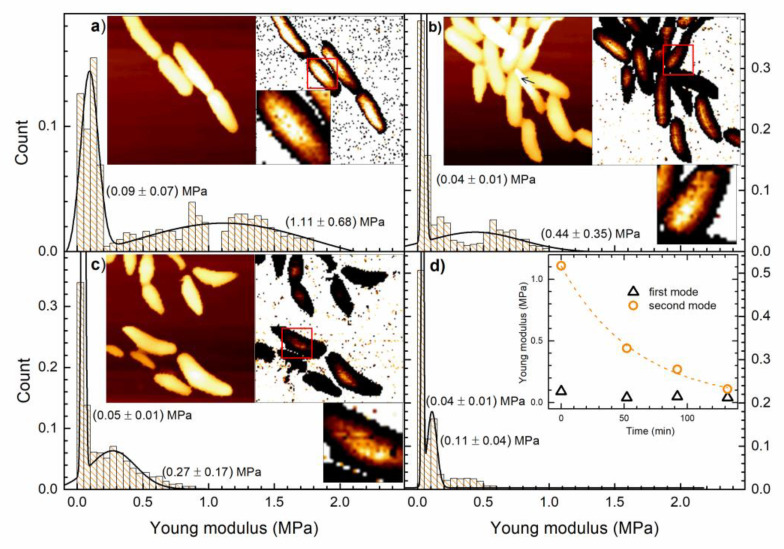
**AFM data of non-treated and AgNPs treated *E. coli* cells at different times**. Topography and YM maps are obtained for the same cell group and the data are collected at different pre- and post-treatment times. Larger images have 128 × 128 resolution, 10 × 10 μm scan size, 0 to 1.5 μm height color scale, and 0 to 1 MPa YM color scale. Histograms, representing count versus Young modulus, are the distributions of the YM data selected within the 5% of the highest points for each scan line. The full lines are the bimodal normal distribution fits with means and the standard deviations given in parentheses. Parts of the YM maps marked with red squares are enlarged. (**a**) Non-treated bacterial cells height data (left image) and the corresponding calculated YM data (right image). (**b**) Treated bacterial cells height data (left image) and the corresponding calculated YM data (right image) whose scan began after 50 min of treatment. (**c**) The same as in (**b**) with the scan beginning after 90 min of treatment (**d**) The same as in (**b**) with the scan beginning after 130 min of treatment.

AFM data acquisition and analysis were used to obtain topography and YM of untreated and AgNP-treated cells. The obtained data are shown in Figure 4. All images have the 128 × 128 resolution, scan size of 10 × 10 micrometers and height color scale up to 1.5 micrometers, while the YM maps have the color scale up to 1 MPa. It takes about 40 min to collect a full AFM data set. For every pixel, the height of the sample is obtained, and the force-distance curve is measured. The YM of each pixel is calculated by fitting those curves. All redundant data points are excluded from the calculations since our interest is only in the YM of the bacterial cell and not of the surrounding substrate. YM data in Figure 4a–c reveal that the substrate/glass is very stiff with some randomly distributed soft points that can be attributed to the Cell-Tak coating.

Figure 4a reveals the AFM topography image of non-treated bacteria given on the left side and the corresponding YM map on the right side. These cells are “the mother cells” of all other cells that are given in Figure 4b–d. It can be noticed that the YM increases as the height of the bacteria increases, i.e., the YM is apparently lower on the cells’ edges.

Due to the oval shape of the cell, the tip of the AFM probe is far from being perpendicular to the cell surface on the edges. Therefore, the Hertz model, for which the calculations of YM are performed, is not valid. Consequently, only data obtained on the top of the cell provide correct information on the bacterial YM. We have extracted and considered only the YM data that correspond to the top 5% of the height points of each scan line. This was done for all obtained YM data sets.

Figure 4a also gives the normalized distribution of the YM values, selected as described above, whose number is found to be 798. The normalized histogram indicates that the YM has a bimodal distribution. The full line is the corresponding bimodal normal distribution fit with the following mean values and standard deviations: (0.09 ± 0.07) MPa and (1.11 ± 0.69) MPa. The enlarged part of the map shows that the softer (darker) points, for which the YM is within (0.09 ± 0.07) MPa, are evenly distributed on the bacterial surface. Rough estimate is that the softer points (first four bins of the histogram) make about 30% of all scanned points.

Figure 4b shows the height and YM map for the AgNPs treated cells. Data collection started about 50 min after the start of the treatment. It can be noted, by comparing the cell morphologies in Figure 4a,b, that the bacterial overall shape and appearance has not changed. In Figure 4b, there are a few cells (denoted by an arrow) that appear to be narrower on one end since this end is loosely attached. This occurs when bacteria stop growing in a monolayer and are stacked on top of each other. The YM data that correspond to these loosely attached parts of bacterial cells were excluded from the histogram. The number of YM points selected for the analysis is 598.

Figure 4b shows that after the treatment, the distribution of YM data remains bimodal, but with modified means and the standard deviations: (0.04 ± 0.01) MPa and (0.44 ± 0.35) MPa. It should be noted that both YM means are now shifted to lower values. The YM data reveal the softening of the bacterial cells. The YM distribution for the softer regions is narrower. These softer regions on top of the cells are evenly distributed and more numerous (when compared to non-treated cells), as can be observed on the enlarged YM map. The rough estimate is that the softer YM population (first two bins of the histogram) now makes about 50% of all YM points.

Figure 4c shows the topography and YM maps obtained for the same cell group. The data acquisition started after 90 min of treatment. By comparing this topography with the topography of untreated cells of Figure 4a, it can be noted that some cells are now shorter and that the YM modulus data reveal further softening of the cells. It can also be seen that some cells are surrounded by some soft matter. The mean values and the standard deviations obtained from the bimodal YM distribution are (0.04 ± 0.01) MPa and (0.27 ± 0.17) MPa, and the number of the used YM points is 834. The softer regions retain the same stiffness, while the stiffer regions become even softer with the sharper distribution. The softer YM population, which makes about 50% of all YM points, remains unchanged. These softer regions of the cell may be observed on the enlarged YM map.

The acquisition of the data presented in Figure 4d began 130 min after the AgNPs have been added to the culture. The means and the standard deviations obtained for the YM histogram are (0.04 ± 0.01) MPa and (0.13 ± 0.24) MPa, and the number of selected YM points is 729. Once again, the softer regions are unaltered, while the stiffer ones become softer. The population of the softer YM points (first bin of the histogram) makes about 50% of all YM points and its distribution sharpens.

The inset of Figure 4d shows the change of the YM mean values with time. The triangles are the YM mean values for softer regions (first mode), while the circles are the YM mean values for stiffer regions (second mode). It can be noted that soon after the beginning of the treatment, the YM of the soft regions drops to half its initial value and then remains constant, while YM of the stiff regions decreases all the time. The dotted line is the exponential fit of the YM data of the stiffer region. The fit is given by the equation $y=1.1e^{-0.02x}$ with *R*^2^ = 0.99.

**Figure 5:**
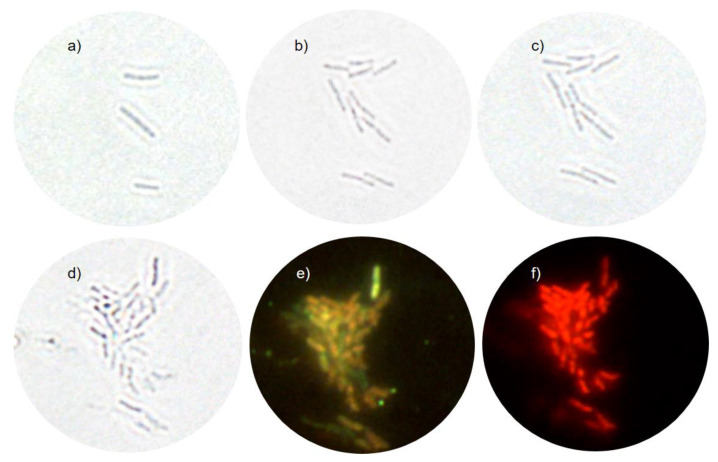
**Optical microscopy data of non-treated and treated *E. coli* cells**. (**a**) Bright-field image of the initial “mother cells” before AFM imaging. (**b**) Bright-field image taken after AFM of non-treated cells, about 1.5 h after the (**a**) image. (**c**) Bright-field image taken after a few minutes of treatment. (**d**) Bright-field image taken after 3 h of treatment. (**e**) SYTO 9 fluorescence image taken after 3 h of treatment. (**f**) PI fluorescence image taken after 3 h of treatment.

Bright-field images were obtained before and after the AgNPs treatment. Figure 5a,b show untreated cells before and after AFM scans, respectively. It is visible that the cells have divided proving that the probe does not interfere with the cells′ viability. Bacteria given in Figure 5a are “the mother cells” to all bacteria in subsequent Figure 5b–f. Figure 5c shows the region of interest immediately after the treatment. Figure 5d, taken after 3 h of treatment, shows that the cell division does not cease immediately after the treatment. The image reveals that the cells’ number has increased sometime during the treatment. Figure 5e,f are the fluorescence signal of SYTO 9 and PI, respectively. SYTO 9 stains all bacterial cells, while the PI signal of bacteria appears only if the membrane is permeabilized. When both dyes are present at once, PI exhibits a stronger affinity towards nucleic acids than SYTO 9, so SYTO 9 is displaced by PI [50]. Figure 5e reveals that the green signal of *E. coli* is reduced, and the signal seems to be shifted towards the yellow. Figure 5f shows that all *E. coli* cells have been permeabilized and every red-colored region of the image belongs to a rod-shaped bacterial cell.

## 4. Discussion

The consistency of *E. coli* cell membrane and bacterial viability were studied upon AgNPs’ treatment to elucidate possible correlation of membrane perturbation with the observed bactericidal effect. The important question is whether the AgNPs permeabilize the cells and if so, how this influences the cell viability. Please note that the PI molecule enters the cells only if they are membrane-permeabilized. After 90 min of the treatment with ½MIC dose, we found that 86% of cells were PI-positive, as shown in Figure 1b. After the same period, the time-killing assay revealed the 80% reduction of the viable-cell population, as shown in the inset of Figure 1a. These results demonstrate that the PI-positive cells are also non-viable and that the membrane permeabilization is closely related to the AgNPs’ antibacterial mode of action. The 90-min treatment at a concentration corresponding to the MIC value induced PI permeabilization for 94% of the bacterial population, i.e., only 6% of the population remained possibly viable, while the time-killing assay indicated less than 0.3% of viable cells. These MIC data reveal that not all non-viable cells are also PI-positive, so we conclude that the permeabilization of the membrane might not be the only killing mechanism.

It was not possible to compare the AFM data with the time-killing assay data at 2MIC since the growing conditions for the two experiments were quite different, due to the impossibility of shaking the AFM sample during incubation. However, Figure 5f clearly shows that after 3 h of treatment all cells (shown in Figure 5e) are PI-positive, which means that scanned cells in Figure 4d were not viable.

It is widely accepted that cell stress is related to the intracellular ROS level. DCFH_2_-DA fluorescence probe, which diffuses through the cell membrane, is used to measure the bacterial ROS creation during the AgNPs treatment. Due to the intracellular esterases, DCFH_2_-DA deacetylates to non-fluorescent but membrane-impermeable DCFH_2_, which reacts with intracellular ROS to produce the fluorescent DCF [27]. This probe was proved to be suitable as a marker for the total ROS production [27] and is the most widely used fluorescent probe for detecting intracellular H_2_O_2_ [52]. However, it is difficult to extract the ROS fluorescence signal from the total signal generated in the sample. There are two main reasons for this. The first is that the cellular ROS must be resolved from the contribution of the probe’s autooxidation signal. The second is the attenuation of the light by absorption and/or scattering on both the nanoparticles and the cells, which are present in the measured sample.

Two fluorescence experiments were performed to obtain the dependence of the bacterial ROS signal *i*_ROS_ (*v*,*t*) on the AgNPs’ concentration and time: first for different concentrations of AgNPs in dyed PBS without bacteria and the second for the same samples but inoculated with bacteria. The results are shown in Figure 2. Figure 2a shows the time dependence of the signal *I*_AO_(*v*,*t*) that appears in the non-inoculated sample, due to the self-oxidation of the probe for different AgNPs concentrations. The signal increases in time as the oxidation progresses. It also decreases with the AgNPs concentration increase due to their absorption/scattering of the light. A similar masking effect for the DCF fluorescence signal has been reported for iron oxide nanoparticles [53] while coated AgNPs were shown to decrease ROS level due to fluorescence quenching and adsorption of the fluorescent dye [28].

To obtain the influence of bacteria on the fluorescence signal, we compare the signal of the inoculated dyed PBS buffer *I*_B_(0, *t*) with the autooxidation signal *I*_AO_(0, *t*) of the non-inoculated dyed PBS buffer. As shown in the inset of Figure 2a, the correlation between the two signals is linear and it can be written in the form:

(1) $$I_{B}\left(0,t\right)=k_{B}I_{AO}\left(0,t\right)+I_{BO}\;,$$

where *k*_B_ = 0.66 is the attenuation coefficient and the intercept *I_B0_* = 46 a.u. is the constant which depends on the time of preparation of the samples and the offset of the instrument. Bacteria attenuate the existing fluorescence signal throughout the absorption/scattering of the light. This correlation (Equation (1)) proves that within the accuracy of the experiment, there is no additional fluorescence signal generated by bacteria, i.e., bacteria in the dyed PBS buffer do not produce ROS.

The nanoparticles act similarly. They absorb and scatter both the incident light, coming from the instrument’s lamp to the probe in the sample, and the fluorescent light emitted by the molecule. It is reasonable to assume that for low concentrations of the AgNPs, the absorption/scattering coefficient is proportional to the concentration *v* so that the intensity of the incident light on its path to the molecule is reduced by the factor $\left(1-k_{i}v\right)$. Similarly, the intensity of the fluorescent light on its path out the sample is reduced by the factor $\left(1-k_{f}v\right)$, where $k_{i}$ and $k_{f}$ are the constants that for a given sample geometry, depend on the wavelength. The UV-visible absorption spectrum of the LAL-synthesized AgNPs exhibits a typical sharp peak at 404 nm due to nanoparticle’s surface plasmon band absorption [45]. The absorption of the fluorescence light, emitted by DCF at λ = 523 nm, is about half the maximum but is still strong enough to produce the observed fluorescence signal reduction.

Under these assumptions, the fluorescence signal which should be obtained for AgNPs concentration *v*,

(2) $$I_{f}\left(v,t\right)=\left(1-k_{f}v\right)\left(1-k_{i}v\right)I_{f}\left(0,t\right)$$

can be related to the fluorescence signal $I_{f}\left(0,t\right)$, obtained for the nanoparticle-free sample. For the low nanoparticle concentrations, the quadratic term in *v* can be neglected. Therefore, Equation (2) assumes a linear form

(3) $$I_{f}\left(v,t\right)=\left(1-k_{NP}v\right)I_{f}\left(0,t\right),$$

where $k_{NP}$ = $k_{f}+k_{i}$.

Taking into account that the measured intensities differ from the actual values by the constant $I_{0}$, which could appear e.g., due to the offset of the instrument, Equation (3) can be rewritten in terms of the measured quantities as

(4) $$I_{AO}\left(v,t\right)-I_{O}=\left(1-k_{NP}v\right)(I_{AO}\left(0,t\right)-I_{O}),$$

where $I_{AO}\left(v,t\right)$ is the measured fluorescence for the non-inoculated samples (see Figure 2a). Equation (4) gives a linear correlation

(5) $$I_{AO}\left(v,t\right)=\left(1-k_{NP}v\right)I_{AO}\left(0,t\right)+k_{NP}vI_{0}$$

between the measured $I_{AO}\left(v,t\right)$ and $I_{AO}\left(0,t\right)$, with the correlation coefficient being a linear function of *v* whose intercept is equal to 1. The assumptions on the role of AgNPs in the dyed PBS buffer, as given in Equation (5), are confirmed by the correlation analysis of the fluorescence data shown in Figure 3a. The slopes and intercepts obtained from the linear correlations given in Figure 3a were proven to have linear dependencies on the nanoparticle concentration, as shown in the inset of Figure 3a.

Therefore, we conclude that the influence of the AgNPs on the autooxidation of the dye is negligible. The decrease in fluorescence signal, which appears when the AgNPs are added to the batch, appears only because of light absorption and scattering by the nanoparticles.

The evolution of the fluorescence signal for the inoculated samples *I*_B_(*v*,*t*) is shown in Figure 2b. The non-treated-bacteria signal starts with the highest intensity and then gradually drops below the signals obtained for the treated cells. For the MIC-treated bacteria, the signal starts with the lowest intensity and is almost constant for about 40 min after which it increases. These results can be explained in terms of the AgNPs’ double effect. They stimulate cells’ ROS creation, which increases the fluorescence signal, but they also absorb and scatter the incident/emitted light which results in signal reduction. The latter applies to both fluorescence signals: the abiotic one $I_{AO}\left(0,t\right)-I_{O}\;$that appears in the sample without the cells and the ROS related signal $i_{ROS}\left(v,t\right)\;$coming from inside the cell. The total measured signal $I_{B}\left(v,t\right)$ is the sum of those two signals multiplied by reduction factors of the nanoparticles and the bacteria and, similarly as for Equation (4), can be written in the form:

(6) $$I_{B}\left(v,t\right)-I_{O}=k_{B}\left(1-k_{NP}v\right)(I_{AO}\left(0,t\right)-I_{O}+i_{ROS}\left(v,t\right)).$$

Please note that the actual and measured values differ for the offset $I_{O}$. From Equation (6) we obtain

(7) $$i_{ROS}\left(v,t\right)=\frac{I_{B}\left(v,t\right)-I_{O}}{k_{B}\left(1-k_{NP}v\right)}-I_{AO}\left(0,t\right)+I_{O}.$$

Knowing the parameters $k_{B},k_{NP}$ and $I_{0}$, and obtaining the fluorescence response of the $I_{AO}\left(0,t\right)$ sample, Equation (7) enables the extraction of the fluorescence signal due to the intercellular ROS from the $I_{B}\left(v,t\right)$ raw data. The parameter $k_{B}=0.66$, which is related to the fluorescence signal attenuation due to the scattering on the bacterial cells, is the correlation coefficient of the fit given in the inset of Figure 2a. The parameter $k_{NP}=1.92$, which refers to the fluorescence signal attenuation due to the scattering/absorption of the AgNPs, is the correlation coefficient of the slopes’ fit given in the inset of Figure 3a. According to Equation (5), the parameter $I_{0}$ is obtained by dividing the correlation coefficients of the linear fits given in the inset of Figure 3a, i.e., $I_{0}=\frac{107}{1.92}=55.8$ a.u.

It is reasonable to presume that the extracted $i_{ROS}\left(v,t\right)$, shown in Figure 3b, is proportional to the level of the cellular ROS which is dose- and time-dependent. Sub-MIC treatments, for which the bacterial population is not terminally threatened, gives the response of metabolically active cells on a chosen time scale. If the ROS production would be the dominant antibacterial mode of action, the ROS level should increase with the AgNPs concentration and the MIC treatment should result in the highest ROS level. The ROS level dependency is given in the inset of Figure 3b, for t=5 min and t=95 min. It can be seen (for t=95 min) that the ROS level increases with the AgNPs concentration up to 2/3 MIC (v=0.20), assumes the maximum, and then suddenly drops for the MIC treatment. ROS level obtained at t=95 min for the MIC treatment is 3 times lower than for 5/6 MIC (v=0.25) treatment. The highest ROS level is significantly (about 3 times) lower than the ROS level developed for the treatment with 0.33 mM H_2_O_2_. Please note that the later time-dependent level of ROS was obtained from Equation (7) for v=0, and is given in double cross-shaped symbols in the inset of Figure 2b.

Altogether, it is reasonable to conclude that the ROS induced oxidative stress is not the predominant antibacterial mode of action of the LAL-synthesized AgNPs against *E. coli*. The ROS level does significantly increase with the AgNPs dose and time, but only for the sub-MIC dose and is still notably lower when compared to the H_2_O_2_ induced ROS level. Our results show that ROS production is related to the antibacterial mode of action for sub-MIC AgNPs treatments, but not for the MIC treatment.

The results on the time evolution of the DCF fluorescence signal in *E. coli* treated with AgNPs [33] are scarce. Published data are raw, which cannot be compared with our ROS data obtained by careful analysis of the fluorescence signal. Interestingly, time dependency of the DCF fluorescence signal given in Figure 2a reveals functional dependency similar to the DCF fluorescence signal of the graphene oxide (GO) and poly (methyl 2-methylpropenoate) (PMMA) fiber mixture [54]. However, the DCF signal of GO/PMMA develops longer lag with the final intensity very close to the H_2_O_2_ DCF signal. Also, according to the baseline data, it seems that the GO/PMMAs mixture does not induce the scattering/absorbance effect as AgNPs do.

To explore the possible treatment-induced alterations, the AgNP-treated bacterial cells were inspected by AFM. The YM was measured since the inspection of the morphology did not give a significant difference between the non-treated and treated cells for all times. We used the QI mode which enables simultaneous imaging and measurement of mechanical properties of bacterial cells [55]. YM was obtained from the force–distance curves by applying the Hertz model for spherical indenters [56].

The YM map of viable non-treated cells, given in Figure 4a, reveals that the periphery of the bacterial cell is apparently more deformable than the apex. This effect occurs due to the curvature of the cell, i.e., the applied force is not normal to the cell [57]. To avoid this artifact the analysis was performed only for the curves obtained from the central region of the cells [58].

The reported average values of YM for untreated *E. coli* vary by several orders of magnitude, as shown in Table 1 in [59]. This might be attributed to the conditions under which the data were obtained such as measurements on the dried bacterial samples. Our YM data are obtained on viable *E. coli* cells in the growth medium. The novel result in our AFM study, which could be of particular interest, is the bimodal distribution of the YM of untreated *E. coli* cells. The distribution has two clearly split maxima that differ in value for an order of magnitude. The softer regions are randomly distributed on the cell surface and cannot be attributed to, for example, an ongoing cell division, which would induce localized YM alterations.

Some findings in the literature could be related to the observed YM variation and inhomogeneity of untreated cells. It is worth noting that DH5α strain is commonly used to maintain and amplify small plasmid DNA [60]. Also, it has been shown that induction of competence for transformation causes pores which can be entry points for macromolecules [61]. Production of calcium competent cells results in pore formation during the heating step [62] and the shockwave transformation causes an increase in YM of dehydrated cells [63]. We speculate that softer parts of the cell could be predestined as the future points of pore formation. However, this hypothesis demands thorough research.

Post-treatment YM histograms, given in Figure 4, reveal the narrowing of the stiffer YM distribution and the shift of its maximum towards the lower values. We presume that the narrowing of the YM distribution is an indicator of metabolic activity reduction. YM map given in Figure 4c, obtained for 90 min of bacterial treatment, reveal softer regions next to the cells. This might be an indicator of the intracellular matter leakage due to cell membrane damage/lysis. It is also worth noting that the YM maximum of stiffer regions decreases exponentially in time with the time constant of about 50 min, as indicated in the inset of Figure 4d. Since the data given in the histogram in Figure 4d are obtained for non-viable cells, it is clear that the cell viability can be related to the softening of the stiffer parts of the cell.

It has been found that the 4 nm SiO_2_ NPs induce the de-structuration of the peptidoglycan layer and lead to the cell lysis [64]. Our results reveal that the mode of antibacterial action of AgNPs is related to the time-dependent softening of the treated cell and possible cell lysis, which might be due to the de-structuration of the peptidoglycan layer.

The treated cells were monitored by the bright-field and fluorescence microscopy to obtain additional cell viability data before and after AFM data acquisition. Figure 5a,b of untreated cells show that AFM imaging does not influence bacterial viability. Figure 5c,d prove that cell growth does not cease immediately upon treatment. This finding is in line with the dynamic nature of the population lag as reported in the model of the AgNP-treated *E. coli* growth [45], which assumes that cells that have not been inactivated continue to grow during treatment. A broad minimum in viable-cell number for the ½MIC treatment (Figure 1a) is also per the model. The fluorescence data in Figure 5e,f reveals that all cells are non-viable after 3 h of treatment.

## 5. Conclusions

The effect of AgNPs treatment on the consistency of the cell membrane and the cell viability is studied through the fluorescence of the PI probe and plating, respectively. The obtained data reveal that PI-positive cells are non-viable, but at the same time, some non-viable bacteria were not PI-positive, leading to the conclusion that permeabilization of the membrane might not be the only killing mechanism.

The change in bacterial ROS level during the AgNPs treatment was obtained from the DCF fluorescence signal. Two experiments were performed to resolve the cellular ROS from the total fluorescence signal: for different concentrations of AgNPs in dyed PBS without bacteria and for the same samples but inoculated with *E. coli* cells. The results showed that the fluorescence signal increases in time but reduces with nanoparticle concentration. Besides the contribution of the probe’s autooxidation effect, these results can be explained in terms of the nanoparticles’ double effect. Specifically, AgNPs stimulate cellular ROS production which increases the fluorescence signal, but they also absorb and scatter the incident/emitted light which results in a signal reduction.

From the linear correlation between the inoculated dyed PBS buffer signal and the autooxidation signal of the non-inoculated dyed PBS buffer, we conclude that non-treated bacteria do not produce ROS. Similarly, from a linear correlation between dyed PBS buffer signals with different AgNPs concentrations, we conclude that the influence of the AgNPs on the autooxidation of the dye is negligible. The decrease in the fluorescence signal, which appears when the AgNPs are added to the non-inoculated batch, appears only because of light absorption and scattering by the nanoparticles.

This analysis enables the extraction of the cellular ROS level from the total fluorescence signal and further conclusions.

The obtained cellular ROS level is dose- and time-dependent. The ROS level does significantly increase with the AgNPs dose and time, but only for the sub-MIC dose and is still about 3 times lower when compared to the H_2_O_2_ induced ROS level. Given the fact that ROS assumes the highest level for the sub-lethal dose, we conclude that the ROS induced oxidative stress is not the predominant antibacterial mode of action of LAL-synthesized AgNPs against *E. coli*.

YM was obtained from the force–distance curves measured at the topmost parts of bacteria. We found that the distribution of the YM data of untreated *E. coli* cells is bimodal, which is particularly interesting. The distribution has two clearly split maxima that differ in value for an order of magnitude. The softer regions are randomly distributed on the cell’s surface.

During the AgNPs treatment, the bacterial YM histograms reveal the narrowing of the stiffer YM maximum and its softening, i.e., shift towards the lower values, which can indicate the metabolic activity reduction. YM data of treated cells also reveal softer regions next to the cells, which might be an indicator of the intracellular matter leakage due to cell lysis. Fluorescence microscopy images of the AFM-inspected AgNPs treated cells have shown that all cells have been PI permeabilized i.e., inactivated.

Bright-field microscopy of the treated cells shows that the cells’ growth does not cease immediately upon treatment. This finding is in line with the dynamic nature of the population lag as reported in the model of the AgNP-treated *E. coli* growth [45], which assumes that cells that have not been inactivated continue to grow.

Altogether, our results indicate that the LAL-synthesized AgNPs mode of antibacterial action includes an increase of the cellular ROS level and destabilization of the cell membrane which includes reduction of its YM and possible cell leakage. We found ROS induced oxidative stress not to be the main killing mechanism.

The bimodal distribution of YM found for the untreated *E. coli* cells is a novel result that could be of general interest in understanding the membrane structure and motivate us for further work.
